# The Indian ban on loose cigarettes

**DOI:** 10.18332/tid/172515

**Published:** 2023-11-09

**Authors:** Jugal Kishore, Jagdish Kaur, Nancy Satpathy, Pratap K. Jena, Epari Venkatarao

**Affiliations:** 1 Department Community Medicine, Vardhman Mahavir Medical College and Safdarjung Hospital, New Delhi, India; 2 Tobacco Free Initiative, World Health Organization Regional Office for South-East Asia, New Delhi, India; 3 Department of Community Medicine, Institute of Medical Sciences and Sum Hospital, Sikhsha ‘O’ Anusandhan University, Bhubaneswar, India; 4 School of Public Health, Kalinga Institute of Industrial Technology, Bhubaneswar, India

**Keywords:** India, loose cigarettes, ban

**Dear Editor,**

India is home to 266.8 million adult tobacco users, with two-thirds of cigarette smokers (21.89 million), one-sixth of bidi smokers (12.09 million), and one-fourth (50.09 million) of smokeless tobacco users buying their products in loose form^1^. Article 16 of the WHO Framework Convention on Tobacco Control (FCTC) aims to prevent minors from accessing tobacco by banning the sale of tobacco products in loose or small quantities. This prohibition is yet to be effectively enforced, as over 70% of tobacco products in India are still sold without proper packaging^1^. Two in five school-going adolescents are purchasing loose cigarettes^2^. Affordability and easy access to loose cigarettes aid in smoking initiation among youths, continued use among smokers, and reduced quit success rate^3^, making it a driver of the smoking epidemic in India. Additionally, the trade of loose cigarettes bypasses the restrictions set by legal provisions related to warning labels on cigarette packs and tobacco taxation. The counterargument for loose cigarettes, such as helping reduce overall cigarette consumption and gradual quitting^4^, is in contrast to the scientific evidence that tobacco in any amount is harmful to health^3^.

India’s current anti-tobacco legislation Cigarettes and Other Tobacco Products Act (COTPA), 2003 is dynamic but does not prohibit the sale of cigarettes individually or in small (<20 cigarettes) packets^5^, as stipulated in Article 16 of the WHO FCTC^6^. However, at the sub-national level, out of 28 States and eight Union Territories (UTs), 16 States or UTs have banned the sale of loose tobacco products, directly or indirectly^4^. The latest recommendation in December 2022 by the Indian Parliamentary Standing Committee on Health and Family Welfare to ban the sale of single sticks of cigarettes has received a lot of attention in the Indian media^7^.

According to the WHO Global Tobacco Control Report, as of 2019, about eight countries had implemented measures to restrict the sale of individual cigarettes^8^. In countries where the sale of single cigarettes is restricted, the tobacco industry tries to circumvent these measures by offering compact packets of cigarettes and other tobacco products that are more affordable and accessible than traditional packs. Singles provide a perverse incentive for vendors to extract additional profits^9^. Singles make it easier for the tobacco industry to promote new brands and do market research on consumers at the point of sale (POS)^10^.

The Pan-India recommendation to ban singles/loose tobacco products is a welcome milestone in India’s tobacco control efforts. Considering the high burden of bidi smoking and smokeless tobacco in India, special focus should be given to bidi and smokeless tobacco retailing. In light of its significance in India’s tobacco control efforts, it is imperative to prioritize the effective implementation of this policy.

## Data Availability

Data sharing is not applicable to this article as no new data were created.
